# Energy Efficiency of User-Centric, Cell-Free Massive MIMO-OFDM with Instantaneous CSI

**DOI:** 10.3390/e24020234

**Published:** 2022-02-03

**Authors:** Tongzhou Han, Danfeng Zhao

**Affiliations:** College of Information and Communication Engineering, Harbin Engineering University, Nangang District, Harbin 150001, China; zhaodanfeng@hrbeu.edu.cn

**Keywords:** cell-free massive MIMO, conjugate beamforming, OFDM, downlink transmission, power control, energy efficiency

## Abstract

In the user-centric, cell-free, massive multi-input, multi-output (MIMO) orthogonal frequency division multiplexing (OFDM) system, a large number of deployed access points (APs) serve user equipment (UEs) simultaneously, using the same time–frequency resources, and the system is able to ensure fairness between each user; moreover, it is robust against fading caused by multi-path propagation. Existing studies assume that cell-free, massive MIMO is channel-hardened, the same as centralized massive MIMO, and these studies address power allocation and energy efficiency optimization based on the statistics information of each channel. In cell-free, massive MIMO systems, especially APs with only one antenna, the channel statistics information is not a complete substitute for the instantaneous channel state information (CSI) obtained via channel estimation. In this paper, we propose that energy efficiency is optimized by power allocation with instantaneous CSI in the user-centric, cell-free, massive MIMO-OFDM system, and we consider the effect of CSI exchanging between APs and the central processing unit. In addition, we design different resource block allocation schemes, so that user-centric, cell-free, massive MIMO-OFDM can support enhanced mobile broadband (eMBB) for high-speed communication and massive machine communication (mMTC) for massive device communication. The numerical results verify that the proposed energy efficiency optimization scheme, based on instantaneous CSI, outperforms the one with statistical information in both scenarios.

## 1. Introduction

Wireless network technology has developed rapidly within the last 30 years, and people’s lives have been profoundly changed by the development of wireless technology [1]. After the concept of Internet of Things (IoT), the number of wireless devices has grown even faster, and the demand for wireless data transmission has continued to increase for years [2]. In order to meet the demand of wireless transmission, scholars have studied massive multi-input, multi-output (MIMO) technology in depth, and cell-free, massive MIMO technology was first proposed in 2015 [3,4,5]. Using the network structure of deploying massive access points (APs) in a specific area, cell-free technology eliminates the cell edge of traditional cellular networks and fundamentally solves the latency problem caused by cell switching. It also solves the coverage problem of cell edges and shadow areas in traditional massive MIMO systems, and provides uniform quality of service (QoS) to users [6,7,8].

In addition to the demand for high data rates, the energy consumption aspect also puts more demands on cell-free massive MIMO technology. The exponential growth of wireless devices not only increases the demand for wireless transmission, but also raises environmental concerns [9,10]; for example, increased carbon emissions and electromagnetic pollution. The deployment of smaller and smaller cellular cells requires additional economic costs and consumes large amounts of power, resulting in higher bills for network operators. The importance of energy efficiency has motivated a large number of scholars to study optimization of the energy efficiency of cell-free massive MIMO. Yang compared the energy efficiency of cell-free and cellular structures for massive MIMO [11]. The energy efficiency of cell-free massive MIMO is significantly higher than that of cellular MIMO due to the advantage of path loss. Ngo analyzes the effect of power consumption overhead of AP, number of antennas, and other factors on the total energy efficiency, using a closed-form expression for spectral efficiency [12]. When the total number of antennas is constant, increasing the number of APs will increase the power of the backhaul link and make the total power increase. Increasing the number of antennas per AP will first increase the spectral efficiency, and if it continues to increase, it will affect the spectral efficiency by reducing the AP distribution density leading to excessive path loss. The method, by which the non-convex constrained conditions in the energy efficiency problem are replaced by first-order Taylor expansions, is proposed to optimize the power control coefficient using a sequential convex approximation (SCA) method to improve the total energy efficiency [13]. Nguyen investigates the energy efficiency optimization problem of zero-forcing (ZF) precoding [14]. By analyzing the network transmission rate and modeling the energy consumption, the energy efficiency analysis of cell-free massive MIMO shows that the energy efficiency is advantageous, while satisfying the QoS requirements.

In addition to the scheme of optimizing energy efficiency by power control coefficients, other schemes for optimization of energy efficiency have been proposed. First-order methods are suitable for solving large-scale problems, and the literature has used first-order methods for solving energy efficiency optimization problems with conjugate beamforming precoding and zero-forcing precoding, respectively, to significantly improve the speed [15,16,17]. Papazafeiropoulos optimizes the pilot reuse factor and AP density to maximize the energy efficiency per square power [18]. An energy efficiency problem is formulated for determining the optimal energy efficiency with respect to the pilot reuse factor, the AP density, and the number of UEs and antennas per AP. The densification of the AP up to a specific value increases the energy efficiency. Jan proposes an energy-efficient AP sleep scheme for millimeter-wave, cell-free massive MIMO networks to address the problem of underutilization of APs during low traffic loads, leading to inefficient utilization and energy deficiency [19]. In addition, the author optimizes the power allocation for maximizing energy efficiency in a user-centric massive MIMO system operating at millimeter-wave frequency [20]. The AP with a hybrid beamforming structure combines with the proposed resource allocation algorithm to effectively improve the energy efficiency and average rate per user. We note that the combination of both user-centric, cell-free networks and millimeter-wave communications is promising because cooperative networks can alleviate the poor connectivity problems suffered by millimeter-wave communications [21,22]. Jiang proposed cell-free massive MIMO combined with orthogonal frequency division multiplexing (OFDM) to resist in frequency-selective fading channels, via subcarrier allocation, in massive machine-type communication (mMTC) scenarios, can simultaneously support up to 3600 terminals’ communication [23]. However, the 95% throughput of each terminal is not high, due to the use of uniform power allocation.

Although the existing research on cell-free massive MIMO continues to grow, a large number of extant cell-free massive MIMO studies directly assume that cell-free technology inherits the channel hardening benefits of massive MIMO, and that good performance can be obtained using simple signal processing [6,7,8,13,17]. The channel hardening phenomenon of massive MIMO makes the small-scale fading disappear gradually with the increase in antenna array size [24,25]. However, it is pointed out in the literature that channel hardening is conditional in the cell-free massive MIMO scheme [26]. Even if the AP is equipped with four antennas, the channel hardening metric of cell-free massive MIMO does not approach that of the system with the large-scale antenna-array. In order to guarantee the performance of cell-free massive MIMO systems in high-speed mobile scenarios, it is assumed that the number of transmitted symbols in coherent time is small [6]. In low-speed mobility scenarios, longer coherence times provide more transmission opportunities. The user equipments (UEs) move more in line with the pedestrian model when the cell-free massive MIMO systems are deployed on campuses, in large venues, and in shopping malls. The literature analyzes that the downlink transmission rate of cell-free massive MIMO, using estimated instantaneous channel state information (CSI), can be substantially increased in slow-varying channels [27].

In this paper, we study the energy-efficient optimization problem of user-centric, cell-free massive MIMO-OFDM in frequency fading channels. Based on the phenomenon that the channel is less hardened in cell-free massive MIMO, we propose to use instantaneous CSI when using energy-efficient optimization. First, we analyze the downlink cell-free massive MIMO-OFDM channel and the downlink available rate of a cell-free massive MIMO-OFDM system. Then, two resource block (RB) allocation schemes are proposed. In the enhanced mobile broadband (eMBB) scenario, each user occupies all RBs to guarantee a higher downlink transmission rate per user; in the mMTC scenario, each terminal occupies one RB to enable the system to support more terminals. Third, we propose downlink energy efficiency optimization to improve the energy efficiency of cell-free massive MIMO-OFDM systems by using instantaneous CSI. Furthermore, the additional energy overhead of CSI exchange between AP and the central processing unit (CPU) is considered in energy efficiency optimization. Finally, we compare the performance of energy-efficiency optimization with different channel information in cell-free massive MIMO-OFDM systems.

The rest of this paper is organized as follows. In Section 2, we describe the user-centric, cell-free massive MIMO-OFDM system model, the channel model, and downlink transmission. In Section 3, we present the RB allocation scheme for two scenarios and the corresponding downlink available rates. The power consumption model and corresponding energy efficiency is presented in Section 4. In Section 5, we modeled the energy efficiency optimization problem over the whole subcarrier and transformed the problem to a simple optimization problem on the subcarrier, which is solved by SCA. We provide numerical results and discussions in Section 6. Finally, we conclude the paper in Section 7.

## 2. System Model

In a user-centric, cell-free massive MIMO-OFDM system, there are *M* APs, with a single antenna, which are distributed randomly in the finite-sized network region, and the APs serve *K* UEs with a single antenna. APs process data from the uplink and downlink in a collaborative manner, which access a CPU via a backhaul link. The user-centric, cell-free massive MIMO-OFDM system contains *N* subcarriers in the downlink, and the bandwidth of each subcarrier is $\Delta_{f}$.

**Figure 1:**
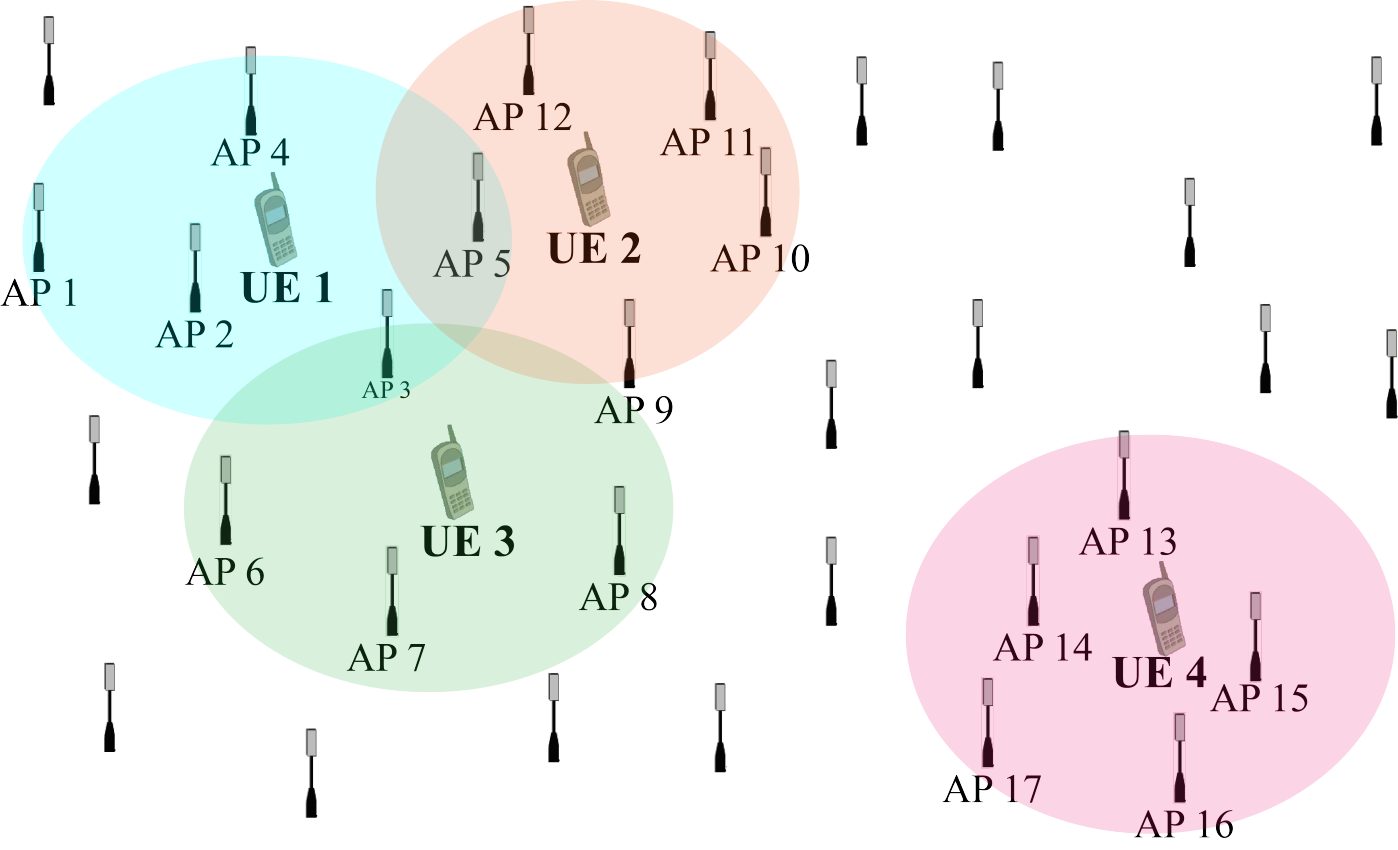
The user-centric cell-free massive MIMO-OFDM scheme.

The user-centric, cell-free massive MIMO-OFDM system assigns a set of APs to each UE. The APs in the set collaborate to send and receive uplink and downlink signals from the UE. As shown in Figure 1, AP 1–5 form $\Omega_{k=1}$ set, and the APs in $\Omega_{k=1}$ serve UE 1 at the same time. Each UE is served by multiple APs to guarantee high-quality communication. Similarly, each AP can also serve multiple UEs at the same time. AP 5 in the figure maintains connections to both UE 1 and UE 2.

The symbol $\Omega_{k}$ is the set which contains the APs serving the UE *k*, and the symbol $\Omega_{m}$ is the set which contains the UEs served by the AP *m*. When $\Omega_{m}$ is an empty set, the AP *m* remains silent. In downlink transmission, the symbol $x_{m,n}$, sent by the AP *m* on the *n*-th subcarrier, are the superposition of the symbols for UEs in $\Omega_{m}$, and the energy of each sent symbol is controlled by the power control coefficients $\eta_{mk,n}$.

### 2.1. Channel Model

The multi-path fading channel is a fading channel caused by multiple signal propagation paths due to reflection and scattering of objects between the transmitter and receiver in wireless communication. The multi-path fading channel between the AP *m* and the UE *k* is modeled as an *L*-tap filter, as follows:

(1) $$\begin{array}{cc} g_{mk}\left[t\right] & ={\left[g_{mk,0}\left[t\right],\ldots ,g_{mk,L-1}\left[t\right]\right]}^{T}\\ \; & ={\left[\sqrt{\beta_{mk}}h_{mk,0}\left[t\right],\ldots ,\sqrt{\beta_{mk}}h_{mk,L-1}\left[t\right]\right]}^{T}\\ \; & =\sqrt{\beta_{mk}}{\left[h_{mk,0}\left[t\right],\ldots ,h_{mk,L-1}\left[t\right]\right]}^{T}\\ \; & =\sqrt{\beta_{mk}}h_{mk}\left[t\right] \end{array}$$

where $\beta_{mk}$ is the large-scale fading and $h_{mk}$ is the small-scale fading. *L* is not smaller than the max delay $T_{d}$. Furthermore, the *l*-th gain, $h_{mk,l}\left[t\right]$, of multiple propagation paths is shown as follows [23,28]:

(2) $$h_{mk,l}\left[t\right]=\sum_{i}a_{i}e^{-j2\pi f_{c}\tau_{i}}sinc\left(l-\frac{\tau_{i}}{T_{s}}\right)$$

where $f_{c}$ is carrier frequency, $a_{i}$ and $\tau_{i}$ are attenuation and delay of the *i*-th propagation path, respectively, and $T_{s}$ is the sampling time of the system.

OFDM converts high-speed serial data streams into *N*-parallel data streams, where each is transmitted on a subcarrier with narrower bandwidth. In another words, it converts a bandwidth *B* into an *N*-parallel narrowband with the bandwidth $\Delta_{f}$, that resists frequency-selective fading, $B=N\Delta_{f}$. The symbol $G_{mk,n}$ denotes the channel coefficient corresponding to the *n*-th subcarrier between the AP *m* and the UE *k*. Furthermore, $G_{mk,n}$ is obtained by the following:

(3) $$G_{mk}=\left[\begin{array}{c} G_{mk,0}\\ \cdots \\ G_{mk,N-1} \end{array}\right]=\left[\begin{array}{ccc} \omega_{N}^{0} & \cdots & \omega_{N}^{0(N-1)}\\ \vdots & \ddots & \vdots \\ \omega_{N}^{(N-1)0} & \cdots & \omega_{N}^{\left(N-1)(N-1\right)} \end{array}\right]\left[\begin{array}{c} g_{mk}\\ 0\\ \vdots \\ 0 \end{array}\right]$$

where $\omega_{N}^{mn}=exp\left(2\pi jmn/N\right)$.

### 2.2. Downlink Transmission

In the frequency domain, the OFDM symbol transmitted at AP, *m*, denotes $X_{m}={\left[X_{m,0},\ldots ,X_{m,N-1}\right]}^{T}$. The transmitted symbol on the *n*-th subcarrier can be expressed in the following form:

(4) $$X_{m,n}=\sum_{k\in \Omega_{m}}X_{mk,n}$$

The symbol $X_{mk,n}$ is the precoded symbol for the UE *k*. In this paper, we consider the conjugate beamforming precoding scheme—$X_{mk,n}=\sqrt{\eta_{mk,n}}G_{mk,n}^{*}s_{k,n}$. Furthermore, $s_{k,n}$ is the symbol for UE *k* on the *n*-th subcarrier. The corresponding OFDM symbol in the time domain is denoted as $x_{m}={\left[x_{m,0},\ldots ,x_{m,N-1}\right]}^{T}$, and $x_{m}$ is *N*-point inverse discrete Fourier transform (IDFT) of $X_{m,n}$. In order to resist the effect of multi-path fading in the wireless channel, the cyclic prefix (CP) is added before the OFDM symbol is sent, with $L_{cp}\geq L$, and the CP is removed after reception. The received signal after the CP is removed is as follows:

(5) $$y_{k}=\sum_{m=1}^{M}g_{\mathrm{mk}}^{N}⊗x_{m}+z_{k}$$

where $g_{\mathrm{mk}}^{N}$ is an *N*-point channel filter formed by padding zeros and $z_{k}∼\mathrm{CN}\left(0,I\sigma_{z_{k}}\right)$. In the frequency domain, the OFDM system is equivalent to an *N*-parallel downlink transmission system, in which a downlink frequency-selective fading wideband channel into the *N*-parallel frequency-flat fading narrowband channels. The downlink channels on each subcarrier are orthogonal to each other, and each subchannel can be represented using a single-tap filter. The frequency domain symbol received on the n-th subcarrier is expressed as follows:

(6) $$\begin{array}{cc} Y_{k,n} & =\sum_{m=1}^{M}G_{mk,n}X_{m,n}+Z_{k,n}\\ \; & =\sum_{m=1}^{M}G_{mk,n}\sum_{k\in \Omega_{m}}\sqrt{\eta_{mk,n}}G_{mk,n}^{*}s_{k,n}+Z_{k,n},n=0,\cdots ,N-1. \end{array}$$

The channel coefficients $G_{mk,n}$ are estimated by the pilot-aided-based minimum mean squared error (MMSE) with overhead $\tau_{p}$ greater than *K*. According to the analysis in the literature, the downlink spectral efficiency (SE) of the *n*-th subchannel for UE *k* is as follows:

(7) $$Se_{k,n}=log_{2}\left(1+\frac{DS_{k,n}^{2}}{UI_{k,n}^{2}+ES_{k,n}^{2}+\sigma_{Z_{k,n}}^{2}}\right),$$

where

(8) $$DS_{k,n}^{2}=P_{ap}{\left(\sum_{m\in \Omega_{k}}\sqrt{\eta_{mk,n}}{\left|{\hat{G}}_{mk,n}\right|}^{2}\right)}^{2},$$

(9) $$UI_{k,n}^{2}=P_{ap}\sum_{k^{\prime }\neq k}^{K}{\left(\sum_{m\in \Omega_{k^{\prime }}}\sqrt{\eta_{mk^{\prime },n}}{\hat{G}}_{mk,n}{\hat{G}}_{mk^{\prime },n}^{*}\right)}^{2},$$

(10) $$ES_{k,n}^{2}=P_{ap}\sum_{k^{\prime }\neq k}^{K}\sum_{k^{\prime }\neq k}^{K}\eta_{mk^{\prime },n}{\left|{\hat{G}}_{mk,n}\right|}^{2}\gamma_{mk,n},$$

and $\gamma_{mk,n}=E\left\{{\tilde{G}}_{mk,n}{\tilde{G}}_{mk,n}^{*}\right\}$ indicates the variance of channel estimation error [27].

In previous studies of cell-free massive MIMO systems, it was assumed that cell-free massive MIMO inherits the channel hardening effect from massive MIMO and use the statistical channel information for downlink transmission. This scheme is simple to implement. However, in the cell-free massive MIMO system, the APs will not be equipped with a large-scale antenna array, so the channel hardening effect is not significant [26,29,30]. The performance loss is large. The user-centric, cell-free massive MIMO has a limited number of APs serving the UE and the channel is more random.

Therefore, in Equation (7), we proposes that using the estimated instantaneous CSI instead of channel statistics in downlink transmission. It has been verified in the literature that the downlink transmission rate is substantially improved.

## 3. Resouce Block Allocation and Downlink Transmitting Rate

In downlink transmission, subcarrier resources are allocated to UEs according to different scenarios. In this section, we consider two scenarios: eMBB and mMTC. The cell-free massive MIMO-OFDM system divides *N* subcarriers into multiple RB, and allocates RBs according to different kinds of service requirements. Referring to RB defined in 5G new radio (NR), we divide 12 subcarriers into a RB, and the bandwidth of each RB is $12\Delta_{f}$ [31]. The eMBB requires very high transmission rate, and the number of UEs in the scenario is much smaller than the number of APs, K<<M. The mMTC supports far more terminals than eMBB—each terminal receives a low total amount of bits in each transmission, and the rate demand is low.

### 3.1. eMBB Case

**Figure 2:**
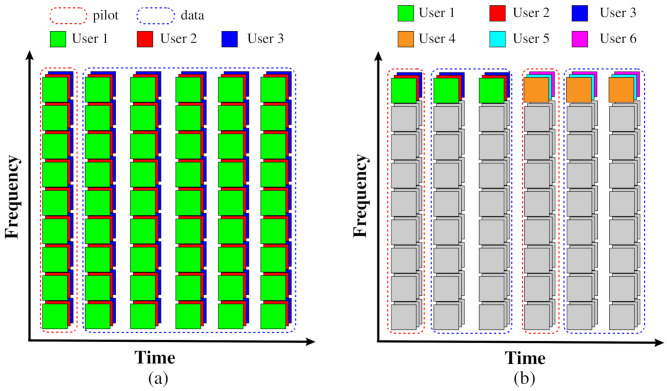
Resource block allocation scheme: (**a**) each UE occupies all RBs for eMBB; (**b**) each UE occupies only one RB for mMTC.

The eMBB is designed to meet the demand for high-speed data transmission. To meet this demand, each UE occupies all subcarriers. The purpose of this allocation scheme is to enable each UE to transmit with maximum bandwidth. Benefiting from the favorable propagation conditions in cell-free massive MIMO, the channels between different UEs are approximately orthogonal. The RB allocation scheme is shown in Figure 2a. A coherence time block consists of the CSI acquisition phase and the data transmission phase.

Considering the overhead of pilot and CP of OFDM, the expression for the downlink transmission rate of the *k*-th UE is as follows:

(11) $$R_{k}=\frac{N_{c}-N_{p}}{N_{c}}\cdot \frac{N}{N+L_{cp}}\sum_{n=0}^{N-1}\Delta_{f}Se_{k,n}$$

where $N_{c}=\left\lfloor \tau_{c}/\left(N+L_{cp}\right)\right\rfloor$ and $N_{p}=\left\lceil \tau_{p}/12\right\rceil$ denote the number of OFDM symbols contained in the coherence time and the number of OFDM symbols occupied by the pilot sequence, respectively. The operators ⌊·⌋ and ⌈·⌉ are floor and ceil function. The pilot sequences are not inserted in all RBs during the pilot transmission phase. Instead, the pilot sequence are inserted at intervals, and the complete channel is recovered by interpolation when the channel estimation is finished. Filling the uplink symbols in other RBs can improve the uplink transmission efficiency. Because we are focused on the downlink transmission in this paper, we do not consider the uplink case.

### 3.2. mMTC Case

To meet the demand for massive terminal transmission, the cell-free massive MIMO-OFDM system assigns multiple UEs to an RB. So, one OFDM symbol can service more UEs. Because the size of packets in mMTC scenario is much smaller than that in the eMBB scenario and the coherence time of the channel is longer in the low-speed mobile scenario, the more UE can be scheduled during a coherence time block. As shown in Figure 2b, for example, user 1,2,3 complete the transmission by occupying 3 OFDM symbols, and more UEs can be scheduled for transmission in the same coherence time.

The downlink transmission rate of each subcarrier assigned to UE *k* is expressed as follows:

(12) $$R_{k,n}=\frac{N_{d}}{N_{d}+N_{p}}\cdot \frac{N}{N+L_{cp}}\Delta_{f}Se_{k,n}$$

where $N_{d}$ is the number of OFDM symbols occupied by the data. The downlink transmission rate of UE *k* is $R_{k}=\sum_{n}R_{k,n}$.

Assuming that each RB is occupied by *q* UEs, the cell-free massive MIMO-OFDM system can support a total of qN/12 UEs transmitting simultaneously.

## 4. Power Consumption Model and Energy Efficiency

The energy consumption of a cell-free massive MIMO-OFDM system consists of two components: (1) the amplifier and basic circuit power consumption of M APs; (2) the power consumption of the backhaul link connecting CPU and APs [13,32,33,34].

(13) $$P_{total}=\underset{\text{Power of APs}}{\underbrace{\sum_{m=1}^{M}P_{m}}}+\underset{\text{Power of backhaul link}}{\underbrace{\sum_{m=1}^{M}P_{bh,m}}}$$

The power consumption $P_{m}$ can be modeled as follows:

(14) $$P_{m}=\frac{P_{ap}}{\alpha_{m}}\sum_{k\in \Omega_{m}}\sum_{n=0}^{N-1}\eta_{mk,i}{\left|{\hat{G}}_{mk,i}\right|}^{2}+P_{tc,m}$$

where $\alpha_{m}$ is the power amplifier efficiency, and $P_{tc,m}$ is the internal power required to run the circuit components of the m-th AP. The power consumption $P_{bh,m}$ of the backhaul link is modeled as follows:

(15) $$P_{bh,m}=P_{0,m}+P_{bt,m}\left(\Delta f\sum_{k\in \Omega_{m}}^{K}\sum_{n=0}^{N-1}R_{k,n}+R_{m,CSI}\right)$$

where $P_{0,m}$ is a fixed power consumption of each backhaul, and $P_{bt,m}$ is the traffic-dependent power consumption. $R_{k,n}$ and $R_{m,CSI}$ are the rate of data transmission and the overhead of updating CSI, respectively. In other studies, the overhead of exchanging CSI between CPU and AP is considered to be negligible. In this paper, we will use instantaneous CSI for power control, and CSI is updated more frequently, so we will consider the overhead of updating CSI between CPU and AP. Furthermore, $R_{m,CSI}$ is computed as follows:

(16) $$R_{m,CSI}=\left\{\begin{array}{c} \frac{\left\lceil N/12\right\rceil \cdot |\Omega_{m}|}{t_{c}},\text{For eMBB},\\ \frac{\left\lceil N/12\right\rceil \cdot |\Omega_{m}|}{t_{d}},\text{For mMTC}, \end{array}\right.$$

where $t_{d}$ is the duration of transmitting a packet and $t_{c}$ is the coherence time. Swapping CSI between the AP and CPU leads to increase load on the fronthaul. Several techniques exist to cope with the higher load in the link. For example, techniques such as non-orthogonal waveforms and non-orthogonal FDM in optical networks [35,36]. It can provide more efficient transmission in capacity-constrained links.

The energy efficiency is defined as the sum throughput, divided by total power consumption in the network, as follows:

(17) $$E_{e}=\frac{\sum_{k=1}^{K}\sum_{n=0}^{N-1}R_{k,n}}{P_{total}}$$

## 5. Energy Efficiency Optimization

Satisfying the transmission rate per user and the AP transmit power constraint, the problem of allocating power coefficients to maximize energy efficiency is described as follows:

(18) $$\begin{array}{cc} \max_{\eta_{mk,n}} & E_{e}\\ s.t. & \sum_{n=0}^{N-1}R_{k,n}\geq {\bar{R}}_{k},\forall k;\\ \; & \sum_{k\in \Omega_{m}}^{K}\sum_{n=0}^{N-1}\eta_{mk,n}{\left|{\hat{G}}_{mk,n}\right|}^{2}\leq N,\forall m;\\ \; & \eta_{mk,n}\geq 0,\forall m,k,n. \end{array}$$

The joint assignment of power coefficients of UEs and subcarriers makes the scale of the problem very large, and, at the same time, the problem is non-convex. It makes the problem difficult to solve. Therefore, in this paper, we will restrict the maximum transmitting power of each UE to be equal on different subcarriers, in order to simplify the difficulty of solving the power coefficients. The power control coefficients of different RBs within the coherent bandwidth can be solved simultaneously, and the power control coefficients of RBs in different coherent bandwidths are solved independently, so that parallelization can be achieved. Therefore, Problem (18) was modified to the following form:

(19) $$\begin{array}{cc} \max_{\eta_{mk,n}} & E_{e}\\ s.t. & R_{k,n}\geq {\bar{R}}_{k,n},\forall k;\\ \; & \sum_{k\in \Omega_{m}}^{K}\eta_{mk,n}{\left|{\hat{G}}_{mk,n}\right|}^{2}\leq 1,\forall m;\\ \; & \eta_{mk,n}\geq 0,\forall m,k. \end{array}$$

In Problem (19), the total transmission rate of each UE is replaced by a constraint on the average transmission rate. By constraining the transmission rate on each subcarrier to be greater than the average transmission rate on each subcarrier, the function of the logarithmic sum is replaced by a logarithmic function. In addition, the total power constraint for all subcarriers is replaced by a power constraint for each subcarrier. Problem (19) is also non-convex, but can be solved with the sequential convex approximation algorithm [13,37].

## 6. Results

To evaluate the energy efficiency performance of the proposed scheme, we consider *M* APs and *K* UEs deployed in a square area of 1000 × 1000 m^2^. In the eMBB scenario, the number of UEs is much smaller than the number of APs; in the mMTC scenario, each UE occupies fewer subcarriers, while the number of UEs being served is much larger than the number of APs. The channel model is the 3rd Generation Partnership Project (3GPP) UMi channel model [38]. The noise variance at the receiver is assumed to be $\sigma_{w}^{2}=T_{0}\times \kappa \times B\times NF$, where κ, *B*, and NF are the Boltzmann constant, bandwidth, and noise figure, respectively. The system contains *N* subcarriers, where every 12 subcarriers are divided into a group as an RB. Other parameters are summarized in Table 1.

### 6.1. Simulation for eMBB

**Figure 3:**
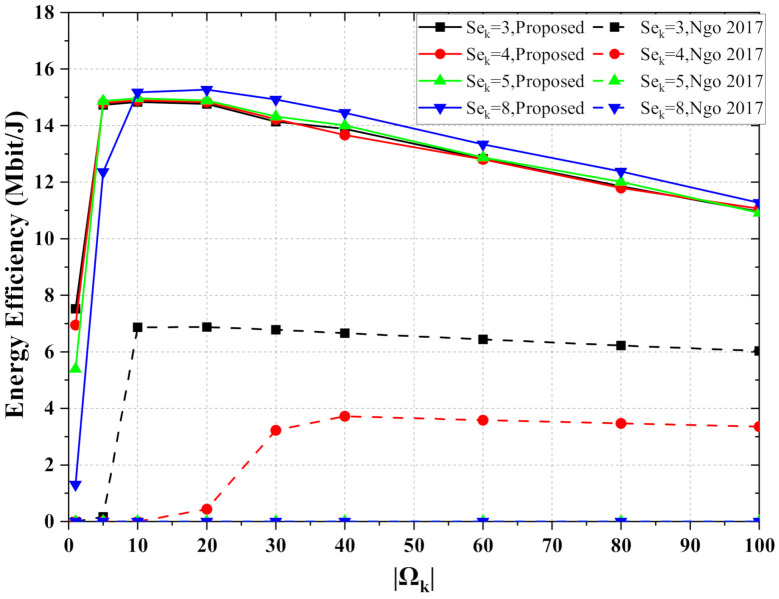
The energy efficiency versus the number of APs serving each UE, where M=100 and K=10.

Figure 3 illustrates the number of APs serving each UE versus energy efficiency performance. The APs servicing UE are selected based on large scale fading. $\left|\Omega_{k}\right|$ is the number of APs serving each UE. In the proposed scheme, the energy efficiency can reach the maximum, about 15 Mbit/J, when each UE is served by 10–20 APs. When UE is served by less than 10 APs, the energy efficiency decreases significantly. For example, when each UE is connected to only 1 AP, the energy efficiency is only half of the optimal value, or even less. Comparing the energy efficiency optimization scheme with the statistical channel information, the proposed scheme has significantly higher energy efficiency. In the Figure 3, the dashed line keeps zero-value when the QoS requirement is not less than 5 bit/s/Hz. The reason is that the power control factor solution fails, due to high QoS requirements during optimization. In contrast, the proposed scheme still maintains high energy efficiency when the QoS requirement is 8 bit/s/Hz. Weak channel hardening makes the channel statistics deviate from the accurate channel information. Just allocating power with statistic channel information leads to significant performance loss.

**Figure 4:**
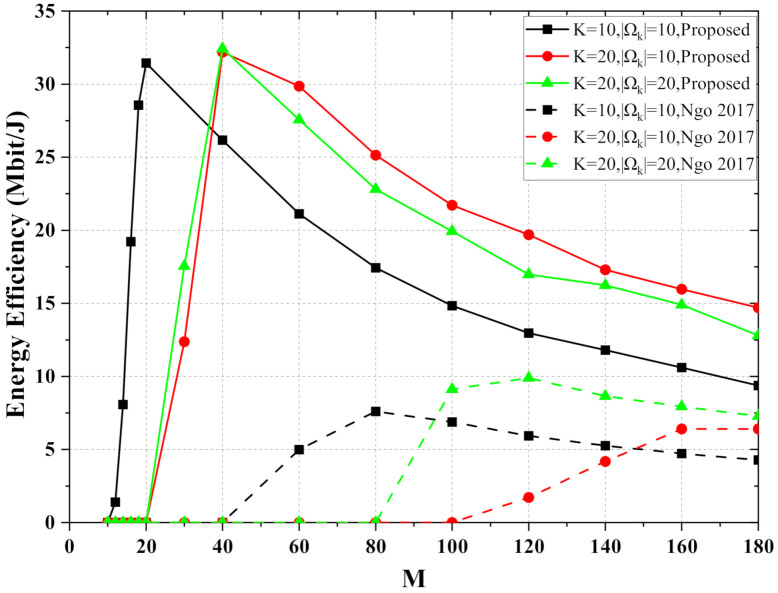
The energy efficiency versus the number of AP, where the required QoS is $Se_{k}=3$ bit/s/Hz.

In Figure 4, we compare the performance of cell-free massive MIMO-OFDM systems for different numbers of APs. As *M* increases, the energy efficiency first increases, then decreases as it approaches the top. When the number of UEs in the scenario increases, more APs need to be deployed to achieve optimal energy efficiency. The energy efficiency performance of the proposed scheme is much higher than that of the scheme with statistic channel information for the same number of APs. When AP = 40, the cell-free massive MIMO-OFDM system serves 20 UEs and meets the QoS requirement of 3 bit/s/Hz in the proposed scheme. However, the scheme with statistical information is unable to meet the communication requirements of 20 UEs or even 10 UEs under the same conditions. While centralized massive MIMO systems benefit from the channel hardening phenomenon, near-optimal performance can be achieved by power control without instantaneous CSI. However, cell-free massive MIMO systems do not fully benefit from channel hardening in the same way. Using only statistical information for allocation leads to a severe performance loss compared with the full utilization of channel information.

### 6.2. Simulation for mMTC

**Figure 5:**
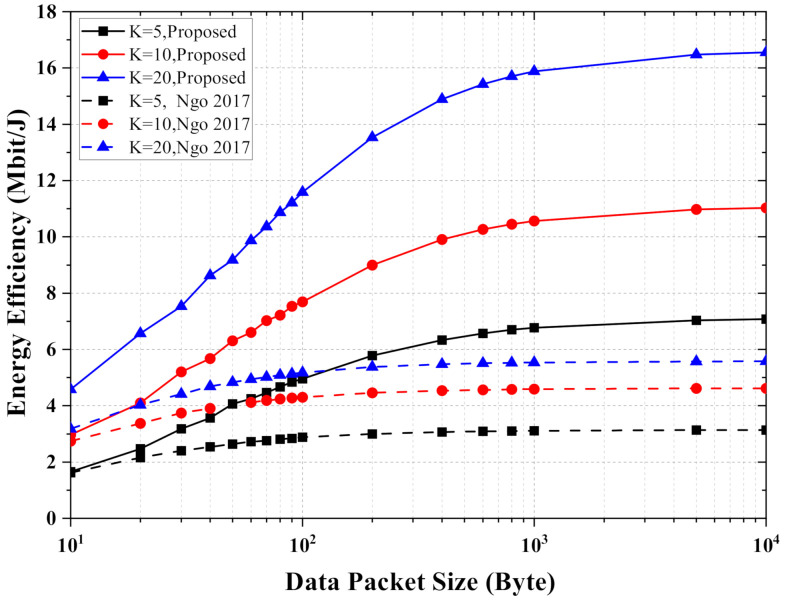
The energy efficiency versus the data packet size, where M=100, $|\Omega_{k}|=10$ and the required QoS is $Se_{k}=3$ bit/s/Hz.

In the mMTC scenario, the 2400 subcarriers are divided into 200 RBs, and we assign multiple terminals to each RB to enable the cell-free massive MIMO-OFDM system for transmitting to more terminals simultaneously. In Figure 5, we compare the energy efficiency performance with 1000 UEs, 2000 UEs, and 4000 UEs. The energy efficiency of both schemes increases as the packet size increases. The performance gap between the scheme using channel statistics information for transmitting small and large packets is smaller than the performance gap of the proposed scheme using instantaneous CSI. In the proposed scheme, the energy efficiency is more severely affected when transmitting small packets, as more channel information exchange takes up the communication opportunity. When transmitting large packets, the energy efficiency is no longer significantly affected by the performance loss due to less channel information exchange. In this case, the advantage of the proposed scheme is more evident.

## 7. Conclusions

Based on the fact that the channel hardening effect of cell-free massive MIMO-OFDM system is no longer obvious, this paper proposes the optimization scheme of energy efficiency of the cell-free massive MIMO-OFDM system by replacing statistical CSI with instantaneous CSI. The effect of channel information exchange between AP and CPU on energy efficiency is also considered. The system uses OFDM modulation and divides 12 subcarriers into one RB. In the eMBB scenario, each UE occupies all RBs for communication; in the mMTC scenario, each UE occupies only one RB to ensure that the system can support more UEs to communicate simultaneously. The simulation results verify that the proposed scheme in both scenarios provides a significantly better performance than the one using channel statistics to optimize energy efficiency.

## Figures and Tables

**Table 1:** Key Simulation Parameters.

Parameter	Value
Area	1000 × 1000 m^2^
Transmit Power of APs and terminals	1 W
Number of APs	10–180
Number of UEs in eMBB	10–20
Number of UEs in mMTC	1000–4000
System bandwidth *B*	18 MHz
Number of subcarriers	2400
Subcarrier bandwidth $\Delta_{f}$	7.5 kHz
Subcarriers in an RB	12
Cyclic prefix length	100
Centering frequency	2.0 GHz
Height of AP and UE antenna	10 m and 1.5 m
Noise figure (NF)	9 dB
κ	1.381 × 10^−23^ J/K
$T_{0}$	290 K

## Data Availability

Not Applicable.

## References

[1] Saad W., Bennis M., Chen M. (2019). A vision of 6G wireless systems: Applications, trends, technologies, and open research problems. IEEE Netw..

[2] Cisco (2020). Cisco Visual Networking Index: Forecast and Trends, 2018–2022.

[3] Marzetta T.L., Ngo H.Q. (2016). Fundamentals of massive MIMO.

[4] Ngo H.Q., Ashikhmin A., Yang H., Larsson E.G., Marzetta T.L. Cell-free massive MIMO: Uniformly great service for everyone. Proceedings of the 2015 IEEE 16th international workshop on signal processing advances in wireless communications (SPAWC).

[5] Nayebi E., Ashikhmin A., Marzetta T.L., Yang H. Cell-free massive MIMO systems. Proceedings of the 2015 IEEE 49th Asilomar Conference on Signals, Systems and Computers.

[6] Ngo H.Q., Ashikhmin A., Yang H., Larsson E.G., Marzetta T.L. (2017). Cell-free massive MIMO versus small cells. IEEE Trans. Wirel. Commun..

[7] Buzzi S., D’Andrea C. (2017). Cell-free massive MIMO: User-centric approach. IEEE Wirel. Commun. Lett..

[8] Nayebi E., Ashikhmin A., Marzetta T.L., Yang H., Rao B.D. (2017). Precoding and power optimization in cell-free massive MIMO systems. IEEE Trans. Wirel. Commun..

[9] Alsharif M.H., Kelechi A.H., Kim J., Kim J.H. (2019). Energy efficiency and coverage trade-off in 5G for eco-friendly and sustainable cellular networks. Symmetry.

[10] Fehske A., Fettweis G., Malmodin J., Biczok G. (2011). The global footprint of mobile communications: The ecological and economic perspective. IEEE Commun. Mag..

[11] Yang H., Marzetta T.L. Energy efficiency of massive MIMO: Cell-free vs. cellular. Proceedings of the 2018 IEEE 87th Vehicular Technology Conference (VTC Spring).

[12] Ngo H.Q., Tran L.-N., Duong T.Q., Matthaiou M., Larsson E.G. Energy efficiency optimization for cell-free massive MIMO. Proceedings of the 2017 IEEE 18th International Workshop on Signal Processing Advances in Wireless Communications (SPAWC).

[13] Ngo H.Q., Tran L.-N., Duong T.Q., Matthaiou M., Larsson E.G. (2018). On the total energy efficiency of cell-free massive MIMO. IEEE Trans. Green Commun. Netw..

[14] Nguyen L.D., Duong T.Q., Ngo H.Q., Tourki K. (2017). Energy efficiency in cell-free massive MIMO with zero-forcing precoding design. IEEE Commun. Lett..

[15] Tran L.-N., Ngo H.Q. First-order methods for energy-efficient power control in cell-free massive mimo. Proceedings of the 2019 IEEE 53rd Asilomar Conference on Signals, Systems, and Computers.

[16] Mai T.C., Ngo H.Q., Tran L.-N. APG Method for Energy-Efficient Power Control in Cell-Free Massive MIMO with Zero-Forcing. Proceedings of the 2020 IEEE Eighth International Conference on Communications and Electronics (ICCE).

[17] Mai T.C., Ngo H.Q., Tran L.-N. (2021). Energy-efficient power allocation in cell-free massive MIMO with zero-forcing: First order methods. Phys. Commun..

[18] Papazafeiropoulos A., Ngo H.Q., Kourtessis P., Chatzinotas S., Senior J.M. (2021). Towards optimal energy efficiency in cell-free massive MIMO systems. IEEE Trans. Green Commun. Netw..

[19] García-Morales J., Femenias G., Riera-Palou F. (2020). Energy-efficient access-point sleep-mode techniques for cell-free mmWave massive MIMO networks with non-uniform spatial traffic density. IEEE Access.

[20] Alonzo M., Buzzi S., Zappone A., D’Elia C. (2019). Energy-efficient power control in cell-free and user-centric massive MIMO at millimeter wave. IEEE Trans. Green Commun. Netw..

[21] Gapeyenko M., Petrov V., Moltchanov D., Akdeniz M.R., Andreev S., Himayat N., Koucheryavy Y. (2018). On the degree of multi-connectivity in 5G millimeter-wave cellular urban deployments. IEEE Trans. Veh. Technol..

[22] Rangan S., Rappaport T.S., Erkip E. (2014). Millimeter-wave cellular wireless networks: Potentials and challenges. Proc. IEEE.

[23] Jiang W., Schotten H.D. (2021). Cell-Free Massive MIMO-OFDM Transmission over Frequency-Selective Fading Channels. IEEE Commun. Lett..

[24] Björnson E., Larsson E.G., Marzetta T.L. (2016). Massive MIMO: Ten myths and one critical question. IEEE Commun. Mag..

[25] Lu L., Li G.Y., Swindlehurst A.L., Ashikhmin A., Zhang R. (2014). An overview of massive MIMO: Benefits and challenges. IEEE J. Sel. Top. Signal Process..

[26] Chen Z., Björnson E. (2018). Channel hardening and favorable propagation in cell-free massive MIMO with stochastic geometry. IEEE Trans. Commun..

[27] Han T., Zhao D. (2021). The Downlink Performance for Cell-Free Massive MIMO with Instantaneous CSI in Slowly Time-Varying Channels. Entropy.

[28] Tse D., Viswanath P. (2005). Fundamentals of Wireless Communication.

[29] Hochwald B.M., Marzetta T.L., Tarokh V. (2004). Multiple-antenna channel hardening and its implications for rate feedback and scheduling. IEEE Trans. Inf. Theory.

[30] Ammar H.A., Adve R., Shahbazpanahi S., Boudreau G., Srinivas K.V. (2021). user-centric, cell-free massive MIMO networks: A survey of opportunities, challenges and solutions. arXiv.

[31] Campos J. (2017). Understanding the 5G NR Physical Layer.

[32] Tombaz S., Vastberg A., Zander J. (2011). Energy-and cost-efficient ultra-high-capacity wireless access. IEEE Wirel. Commun..

[33] Zuo J., Zhang J., Yuen C., Jiang W., Luo W. (2016). Energy-efficient downlink transmission for multicell massive DAS with pilot contamination. IEEE Trans. Veh. Technol..

[34] Dai B., Yu W. (2016). Energy efficiency of downlink transmission strategies for cloud radio access networks. IEEE J. Sel. Areas Commun..

[35] Zhou J., Qiao Y., Yang Z., Cheng Q., Wang Q., Guo M., Tang X. (2017). Capacity limit for faster-than-Nyquist non-orthogonal frequency-division multiplexing signaling. Sci. Rep..

[36] Xu T., Xu T., Darwazeh I. (2021). Deep intelligent spectral labelling and receiver signal distribution for optical links. Opt. Express.

[37] Beck A., Ben-Tal A., Tetruashvili L. (2010). A sequential parametric convex approximation method with applications to nonconvex truss topology design problems. J. Glob. Optim..

[38] 3GPP (2018). Study on channel model for frequencies from 0.5 to 100 GHz. 3rd Generation Partnership Project (3GPP).

